# Postbiotics Derived from *Lactococcus lactis* and *Streptococcus thermophilus* Attenuate Experimental Periodontitis by Modulating Macrophage Polarization and Osteoclastogenesis

**DOI:** 10.3390/nu17162638

**Published:** 2025-08-14

**Authors:** Hyun-Joo Park, Mi-Kyoung Kim, Soon Chul Heo, Dong Ki Hong, Soo-Dong Park, Yeon Kim, Soo-Kyung Bae, Hyung Joon Kim, Moon-Kyoung Bae

**Affiliations:** 1 Department of Oral Physiology, School of Dentistry, Pusan National University, Yangsan 50612, Republic of Korea; 2 Dental and Life Science Institute, School of Dentistry, Pusan National University, Yangsan 50612, Republic of Korea; 3 R&BD Center, hy Co., Ltd., Yongin-si 17086, Republic of Korea; 4 Department of Dental Pharmacology, School of Dentistry, Pusan National University, Yangsan 50612, Republic of Korea

**Keywords:** heat-killed probiotics, postbiotics, periodontitis, macrophage polarization, osteoclastogenesis

## Abstract

**Background/Objectives**: The potential of probiotics and postbiotics as adjunctive or alternative therapies for periodontal disease, which is characterized by chronic inflammation and alveolar bone loss, is gaining increasing attention. In this study, we aimed to elucidate the impact of postbiotic *Lactococcus lactis* HY449 and *Streptococcus thermophilus* HY9012 on key cellular processes implicated in the pathogenesis of periodontitis. **Methods**: THP-1 cells were polarized into M1 macrophages by exposure to *Porphyromonas gingivalis* lipopolysaccharide in the presence of postbiotics, i.e., heat-killed forms of HY449 or HY9012. The effect of postbiotics on the differentiation of bone marrow-derived macrophages into osteoclasts was analyzed using tartrate-resistant acid phosphatase staining. An in vivo mouse model of ligature-induced periodontitis was used to assess changes in periodontal tissues. **Results**: The combination of postbiotic *L. lactis* HY449 and *S. thermophilus* HY9012 synergistically modulated macrophage polarization by significantly suppressing pro-inflammatory M1 markers and enhancing anti-inflammatory M2 markers. Additionally, postbiotic HY449 and HY9012 inhibited osteoclast differentiation, downregulating the expression of key osteoclastogenic genes and master transcription factors of osteoclast differentiation. In a mouse model of ligature-induced periodontitis, co-treatment with postbiotic HY449 and HY9012 demonstrated synergistic effects in reducing alveolar bone loss. **Conclusions**: The present findings support the use of postbiotic HY449 or HY9012 as adjunct treatments for the management of periodontitis.

## 1. Introduction

Periodontitis is a severe and advanced form of periodontal disease characterized by the destruction of tooth-supporting structures, including the periodontal ligament and alveolar bone [1]. It is a chronic inflammatory process involving a complex interplay of microbial, inflammatory, and immune responses that ultimately leads to tissue destruction and bone loss [2]. With growing evidence associating periodontitis with systemic diseases, such as diabetes, cardiovascular disease, and rheumatoid arthritis, it has become even more urgent to develop appropriate pharmacological intervention strategies [3]. These strategies should focus on addressing bacterial biofilm formation, modulating host immune responses, and preventing bone resorption [4].

Macrophages play a crucial role in the immune response and tissue remodeling in periodontitis [5]. Their function is influenced by their polarization state, which is generally categorized as either M1 (pro-inflammatory) or M2 (anti-inflammatory). They participate in the destructive and repair stages of periodontal disease [6]. Osteoclasts are multinuclear bone-resorbing cells that originate from the fusion of hematopoietic monocyte/macrophage lineage cells and contribute to bone remodeling [7]. Dysregulated osteoclastogenesis, including the abnormal production and excessive activation of osteoclasts during periodontitis, plays a pivotal role in the destruction of alveolar bone, leading to tooth mobility and eventual tooth loss [8]. Recently, the development of biomodulators that influence macrophage polarization or osteoclastogenesis has emerged as an innovative approach for periodontal therapy [9].

Probiotics and postbiotics have also emerged as promising adjunctive treatment for periodontitis by modulating the oral microbiota, producing antimicrobial substances, and regulating host immune response [10,11]. *Lactococcus lactis* and *Streptococcus thermophilus* are probiotic, facultative, gram-positive anaerobic bacteria [12,13]. They have been shown to block adhesion of cariogenic bacteria to the tooth surface, exert antimicrobial activity against periodontopathogens, and exhibit anti-inflammatory properties against human peripheral blood mononuclear cells [12,14,15]. Despite the promising antimicrobial properties of *L. lactis* HY449 and *S. thermophilus* HY9012 [12,14], their immunomodulatory capacity in regulating host inflammatory responses and bone remodeling processes remains unclear.

Due to limited colonization and safety concerns of live probiotics, postbiotics including heat-killed strains offer a safer and more stable therapeutic alternative for periodontal disease [11]. This study aimed to elucidate the effect of the postbiotics—specifically, heat-killed *L. lactis* HY449 (HK-HY449) and *S. thermophilus* HY9012 (HK-HY9012)—on macrophage polarization and osteoclast differentiation in vitro, and to assess their therapeutic efficacy in experimental periodontitis models in vivo.

## 2. Materials and Methods

### 2.1. Reagents and Antibodies

Recombinant receptor activator of nuclear factor κB ligand (RANKL) and macrophage colony-stimulating factor (M-CSF) were obtained from PROSPEC (East Brunswick, NJ, USA) and PeproTech (Rocky Hill, NJ, USA), respectively. A tartrate-resistant acid phosphatase (TRAP) staining kit was obtained from Sigma-Aldrich (St. Louis, MO, USA). PerCP/Cyanine5.5-conjugated CD80 and CD86 antibodies were obtained from BioLegend (San Diego, CA, USA). Antibodies against human extracellular signal-regulated kinase (ERK), phospho-ERK, p38 mitogen-activated protein kinase (MAPK), phospho-p38 MAPK, c-Jun N-terminal kinase (JNK), phospho-JNK, AKT, phospho-AKT, STAT1, phospho-STAT1, STAT6, and phospho-STAT6 were purchased from Cell Signaling Technology (Danvers, MA, USA). Antibodies against c-Fos and nuclear factor of activated T-cells cytoplasmic 1 (NFATc1) were obtained from Santa Cruz Biotechnology (Dallas, TX, USA). The β-actin antibody was purchased from Bioworld Technology (St. Louis Park, MN, USA). Horseradish peroxidase-conjugated goat anti-rabbit and anti-mouse IgG antibodies were purchased from Thermo Fisher Scientific (Waltham, MA, USA).

### 2.2. Preparation of Postbiotics

Postbiotic *L. lactis* HY449 (HK-HY449) and *S. thermophilus* HY9012 (HK-HY9012) were obtained from hy Co., Ltd. (Yongin, Republic of Korea). The culture media of the two strains were individually subjected to thermal inactivation at 121 ± 6 °C for 20 ± 10 min. The inactivated preparations were subsequently lyophilized and provided in powder form. The postbiotics were resuspended in phosphate-buffered saline (PBS) for use in both in vitro and in vivo experiments. In all experiments, HK-HY449, HK-HY9012, and a 1:1 mixture of the two strains were used.

### 2.3. MTT Assay

Cell viability following treatment with HK-HY449 and HK-HY9012 was evaluated using the MTT assay. Cells were seeded in 24-well plates and exposed to varying multiplicities of infection (MOIs of 1–200) of HK-HY449, HK-HY9012, or vehicle control for up to 3 days. At the indicated time points, MTT solution (5 mg/mL in PBS) was added to each well, followed by a 3 h incubation. The resulting formazan crystals were solubilized in DMSO, and absorbance was measured at 570 nm using a microplate reader.

### 2.4. Macrophage Polarization

Human monocytic THP-1 cells were purchased from the Korea Cell Line Bank (Seoul, Republic of Korea) and grown in RPMI 1640 medium (Gibco, Billings, MT, USA) with 10% fetal bovine serum (Gibco), 1% penicillin–streptomycin (Gibco), and 5 μg/mL Plasmocin^®^ (Invitrogen, Carlsbad, CA, USA). THP-1 cells were differentiated into macrophages by treatment with 150 nM phorbol 12-myristate 13-acetate (PMA; Sigma-Aldrich) for 24 h. To obtain M1 macrophages, M0 macrophages were incubated with 1 μg/mL *Porphyromonas gingivalis* lipopolysaccharide (LPS) (InvivoGen, San Diego, CA, USA) and 20 ng/mL interferon (IFN)-γ (R&D Systems, Minneapolis, MN, USA) and were collected at the indicated time points. For M2 macrophage polarization, M0 macrophages were treated with 20 ng/mL interleukin (IL)-4 and 20 ng/mL IL-13 (R&D Systems). The cells were grown at 37 °C in a humidified environment composed of 95% air and 5% CO_2_.

### 2.5. In Vitro Osteoclast Differentiation

To obtain osteoclast precursors, bone marrow-derived macrophages (BMMs) were isolated from 5-week-old ICR mice. Bone marrow cells were isolated by flushing the femurs and tibiae with α-MEM using a syringe. The harvested cells were treated with red blood cell lysis buffer to remove erythrocytes. The cells were then plated in culture dishes and incubated overnight with α-MEM, 10% fetal bovine serum, and 1% antibiotics. The following day, non-adherent cells were transferred to Petri dishes and cultured in medium supplemented with 30 ng/mL M-CSF for 3 days. Adherent cells were then detached using a mechanical scraper and used as osteoclast precursors. For osteoclastogenesis, BMMs were incubated in osteoclastogenic medium (culture medium supplemented with 30 ng/mL M-CSF and 100 ng/mL RANKL) for 5 days. The medium was replaced every 2–3 days. After 5 days of incubation, osteoclast differentiation was assessed using TRAP staining.

### 2.6. Reverse Transcription-Quantitative PCR (RT-qPCR)

Total RNA was isolated using a RiboEx kit (GeneAll, Seoul, Republic of Korea) and reverse-transcribed using a reverse transcription kit (Promega, Madison, WI, USA). Real-time PCR was performed using SYBR Green premix (Enzynomix, Daejeon, Republic of Korea). Cycling parameters included one cycle at 95 °C for 10 min, followed by amplification for 40 cycles at 95 °C for 15 s, 60 °C for 60 s, and 72 °C for 7 s; the process was performed in a thermocycler (Applied Biosystems, Foster City, CA, USA). The primer sequences used for real-time PCR were as follows: mouse β-actin, 5′-TCTGGCACCACACCTTCTAC-3′ and 5′-TACGACCAGAGGCATACAGG-3′; mouse TRAP, 5′-CGACCATTGTTAGCCACATACG-3′ and 5′-TCGTCCTGAAGATACGGTT-3′; mouse cathepsin K (CTK), 5′-ATATGTGGGCCACCATGAAAGTT-3′ and 5′-TCGTTCCCCACAGGAATCTCT-3′; mouse dendritic cell-specific transmembrane protein (DC-STAMP), 5′-GGGTGCTGTTTGCCGCTG-3′ and 5′-CGACTCCTTGGGTTCCTTGCT-3′; mouse RANKL, 5′-AGGCTGGGCCAAGATCTCTA-3′ and 5′-GTCTGTAGGTACGCTTCCCG-3′; human β-actin, 5′-ACTCTTCCAGCCTTCCTTCC-3′ and 5′-TGTTGGCGTACAGGTCTTTG-3′; human CD80, 5′-CGGAGGCAGGGAACATCACC-3′ and 5′-TGACCACAGGACAGCGTTGC-3′; human CD86, 5′-TCGTGATGGCCTTCCTGCTC-3′ and 5′-TGCAAATTGGCAAGGCAGGT-3′; human CD163, 5′-TGGCATGCAAGCAAC TGGGA-3′ and 5′-GCAGGTTCATGTCCCTGGCA-3′; human CD206, 5′-GCCGTATGCCGGTCACTGTT-3′ and 5′-AGGTCACCGCCTTCCTTCCT-3′; human IL-6, 5′-CTAGAGTACCTCCAGAACAGATTTGA-3′ and 5′-TCAGCAGGCTGGCATTT-3′; human IL-1β, 5′-GACCTGGACCTCTGCCCTCT-3′ and 5′-CTGCCTGAAGCCCTTGCTGT-3′; human tumor necrosis factor (TNF)-α, 5′-CAGGCGGTGCTTGTTCCTCA-3′ and 5′-AGTGCAGCAGGCAGAAGAGC-3′; and human IL-10, 5′-TTAAGGGTTACCTGGGTTGC-3′ and 5′-TTCACGTGCTCCTTGATGTC-3′.

### 2.7. Western Immunoblot Assay

Approximately 30 μg of total protein per lane was loaded on 10% bis-acrylamide gels, and electrophoresis was performed at 80 V for 2 h. The separated proteins were transferred to nitrocellulose membranes (Amersham Pharmacia Biotech, Uppsala, Sweden). The membranes were blocked in 5% skim milk for 1 h under agitation and probed with primary antibodies for 16 h at 4 °C. Next, the membranes were washed three times for 10 min each with PBS and incubated for 1 h at room temperature with horseradish peroxidase-conjugated goat anti-rabbit IgG or goat anti-mouse IgG secondary antibodies. The signal was developed using an enhanced chemiluminescence solution (Amersham Pharmacia Biotech) and visualized using an Azure 300 imaging system (Azure Biosystems, Dublin, CA, USA).

### 2.8. Flow Cytometry Analysis

M0 macrophages were polarized into the M1 or M2 phenotype in the presence or absence of HK-HY449, HK-HY9012, or their combination. After 48 h of polarization, M1 macrophages were incubated with PerCP/Cyanine5.5-conjugated anti-CD86 antibodies (2 μg/mL), and M2 macrophages with PerCP/Cyanine5.5-conjugated anti-CD206 antibodies (2 μg/mL) for 1 h at 4 °C. The cells were then washed twice with PBS and analyzed by flow cytometry (BD Biosciences, Franklin Lakes, NJ, USA), and the mean fluorescence intensity (MFI) was quantified using FlowJo software v10.8.1 (Tree Star, Ashland, OR, USA).

### 2.9. Enzyme-Linked Immunosorbent Assay (ELISA)

Cytokine secretion in conditioned medium was analyzed using ELISA kits according to the manufacturer’s instructions. IL-6, IL-1β, and TNF-α were measured with a human pro-inflammatory cytokine multiplex ELISA kit (Arigo Biolaboratories, Hsinchu, Taiwan), while IL-10 was quantified using a separate ELISA kit (ABclonal, Woburn, MA, USA). Absorbance was recorded at 450 nm using a microplate reader (BIOAND, Guri, Republic of Korea for multiplex; Dynex for IL-10) and cytokine concentrations were calculated by interpolating sample values onto standard curves generated according to the respective protocols.

### 2.10. Ligature-Induced Periodontitis in Mice

Animal treatment and husbandry were performed in accordance with the principles of laboratory animal care. Animal experiments were performed using protocols approved by the Pusan National University Institutional Animal Use and Care Committee (PNU-2024-0401). C57BL/6J wild-type male mice (6 weeks old; 22–25 g) were purchased from Orient Co., Ltd. (Gapyeong, Republic of Korea). Mice were divided into five groups: a negative control group, a ligature-induced periodontitis (LIP) + vehicle group, LIP + HK-HY449 group, LIP + HK-HY9012 group, and LIP + combined HK-HY449 and HK-HY9012 group. Each group consisted of four mice housed together in a single cage. Ligature-induced periodontitis was achieved as previously described [7]. Briefly, the ligatures were placed around the left maxillary second molars using a 5–0 silk suture (AILEE Inc., Busan, Republic of Korea) with a surgical knot. One day after ligature placement, the animals were administered daily oral gavage of HY449, HY9012, or both (1 × 10^9^ cells) for 14 days [16] and the maxillae were collected on day 14. The maxillae were fixed with 4% paraformaldehyde and subjected to micro-computed tomography (MicroCT.SMX-90 CT; Shimadzu Corp., Kyoto, Japan). Images were captured under a rotation of 360°, with an intensity of 90 kV and 100 mA, and reconstituted using Analyze 12.0 (AnalyzeDirect, Inc., Overland Park, KS, USA). Sagittal sections of the maxillary second molars were obtained using the apex of the mesiobuccal and distobuccal roots as references. The level of bone resorption was calculated as the distance from the palatal and mesiobuccal CEJ to the ABC. RNA was isolated from mouse gingival tissues using the RNeasy kit (Qiagen GmbH, Hilden, Germany) according to the manufacturer’s instructions.

### 2.11. Statistical Analysis

Data are represented as mean ± standard deviation of at least three independent experiments. Statistical comparisons were performed using Student’s *t*-test for pairwise group comparisons. All analyses were conducted using IBM SPSS v27 (Chicago, IL, USA), and a *p*-value < 0.05 was considered statistically significant.

## 3. Results

### 3.1. HK-HY449 and HK-HY9012 Alter M1/M2 Marker Expression During Macrophage Polarization

**Figure 1:**
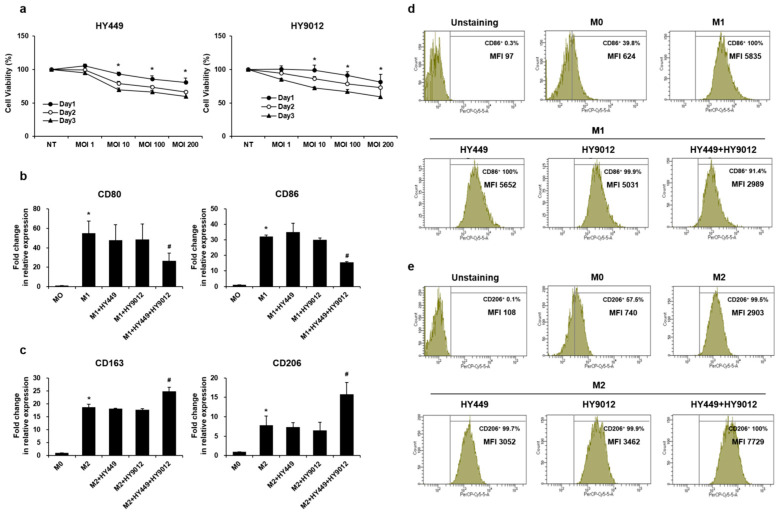
Effect of HK-HY449 and HK-HY9012 on the polarization of M1 and M2 macrophages. (**a**) THP-1 cells were differentiated into M0 macrophages by treatment with 150 nM PMA for 24 h, followed by incubation with varying MOIs of HK-HY449 and HK-HY9012. Cell viability was determined by the MTT assay daily over a period of 3 days. (**b**,**c**) THP-1 cells were exposed to M1 differentiation medium (1 μg/mL *P. gingivalis* LPS and 20 ng/mL IFN-γ) or M2 differentiation medium (20 ng/mL each of IL-4 and IL-13), either alone or in combination with HK-HY449 and 6-HY9012, for 48 h. The mRNA levels of CD80, CD86, CD163, and CD206 were analyzed using RT-qPCR. Expression of the control was set to 1, and values were normalized to β-actin mRNA. (**d**,**e**) Cell surface expression of CD86 and CD206 was measured by flow cytometry, quantifying both the percentage of positive cells and mean fluorescence intensity (MFI). All quantitative data are presented as mean ± standard deviation; * *p* < 0.05 vs. M0, ^#^ *p* < 0.05 vs. M1.

To investigate the potential roles of HK-HY449 and HK-HY9012 in macrophage polarization, we first assessed their effects on the viability of THP-1-derived M0 macrophages. Human THP-1 monocytes were differentiated into stable M0 macrophages by treatment with PMA and subsequently exposed to HK-HY449 or HK-HY9012 at 1–200 MOIs. Cell viability was monitored over 3 days using the MTT assay. As shown in Figure 1a, neither HK-HY449 nor HK-HY9012 induced significant cytotoxicity at MOI = 1, whereas higher MOIs (≥10) resulted in a time-dependent loss of viability. Accordingly, MOI = 1 was selected for all subsequent polarization experiments. We then assessed how HK-HY449, HK-HY9012, and their mixture at a 1:1 ratio affected macrophage polarization toward M1 and M2 phenotypes. M0 macrophages were stimulated with *P. gingivalis* LPS and IFN-γ to induce M1 polarization, or with IL-4 and IL-13 to induce M2 polarization, in the presence of HK-HY449, HK-HY9012, or their combination. As expected, mRNA expression of CD80 and CD86, prototypical M1 markers, was significantly elevated in M1-polarized macrophages compared to M0 controls. While HK-HY449 or HK-HY9012 alone had minimal effects, their combination exerted a synergistic effect, markedly reducing the expression of these markers (Figure 1b). Conversely, the expression of CD163 and CD206 (canonical M2 markers) increased substantially under M2-polarizing conditions. Notably, co-treatment with HK-HY449 and HK-HY9012 further amplified this response, whereas individual treatments had little effect on M2 polarization (Figure 1c). Consistent with these transcriptional changes, flow cytometric analysis revealed that co-treatment with HK-HY449 and HK-HY9012 significantly reduced surface expression of CD86 in M1 macrophages (Figure 1d) and enhanced CD206 expression in M2 macrophages (Figure 1e).

### 3.2. HK-HY449 and HK-HY9012 Differentially Modulate M1/M2 Cytokine Expression and Polarization-Associated Signaling Pathways

**Figure 2:**
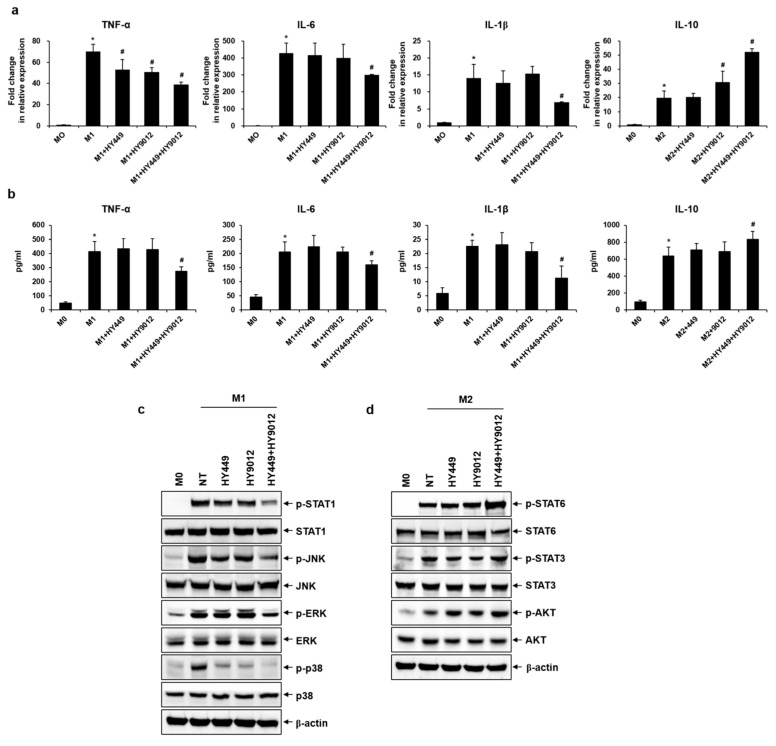
Effect of HK-HY449 and HK-HY9012 on inflammatory cytokine secretion and polarization-associated signaling pathways in M1 and M2 macrophages. THP-1 cells were exposed to M1 or M2 differentiation medium alone or in combination with HK-HY449 and HK-HY9012 for 48 h. (**a**) The mRNA levels of TNF-α, IL-6, IL-1β, and IL-10 were analyzed by RT-qPCR. Expression of the control was set to 1, and values were normalized to β-actin mRNA. (**b**) TNF-α, IL-6, IL-1β, and IL-10 concentrations in cell culture medium were determined by ELISA. All quantitative data are presented as mean ± standard deviation; * *p* < 0.05 vs. M0, ^#^ *p* < 0.05 vs. M1. THP-1 cells were polarized toward the M1 or M2 phenotype for 24 h, during which HK-HY449, HK-HY9012, or their combination was administered for the final hour of the differentiation process. (**c**,**d**) Western blots probed with anti-STAT1, anti-phospho-STAT1, anti-JNK, anti-phospho-JNK, anti-ERK, anti-phospho-ERK, anti-p38, anti-phospho-p38, anti-STAT6, anti-phospho-STAT6, anti-STAT3, anti-phospho-STAT3, anti-AKT, and anti-phospho-AKT antibodies. Anti-β-actin was used as a loading control.

M1 and M2 macrophages are functionally distinct subsets characterized by differential cytokine profiles [17]. M1 macrophages produce pro-inflammatory cytokines, such as TNF-α, IL-6, and IL-1β, whereas M2 macrophages secrete anti-inflammatory cytokines such as IL-10, which contribute to inflammation resolution and tissue repair. To assess whether HK-HY449 and HK-HY9012 modulated these functional phenotypes, we examined their effects on cytokine expression during macrophage polarization. As shown in Figure 2a, TNF-α, IL-6, and IL-1β mRNA levels were strongly upregulated in M1 macrophages. Although treatment with HK-HY449 or HK-HY9012 alone had minimal suppressive effects, their combined administration markedly attenuated the expression of these pro-inflammatory cytokines. In M2 macrophages, IL-10 expression was modestly elevated by individual treatments, while a 1:1 mixture of HK-HY449 and HK-HY9012 had an additive effect. ELISA confirmed the agreement between expression and secretion of TNF-α, IL-6, IL-1β, and IL-10 (Figure 2b). STAT1 is a key transcription factor activated by IFN-γ and plays a central role in promoting M1 macrophage polarization [18]. In addition, MAPK pathways, including JNK, ERK, and p38 MAPK, are primarily activated by microbial stimuli such as LPS and contribute to the induction of pro-inflammatory mediators [19,20]. Based on these mechanisms, we examined whether HK-HY449 and HK-HY9012 modulated the activation of STAT1 and MAPK pathways in M1-polarized macrophages. As shown in Figure 2c and Supplementary Figure S1a, M1 polarization increased the phosphorylation of STAT1, JNK, ERK, and p38. Treatment with HK-HY449 or HK-HY9012 partially attenuated this phosphorylation, whereas co-treatment resulted in a more pronounced suppressive effect. In contrast, STAT3, STAT6, and AKT signaling pathways regulate M2 macrophage polarization associated with anti-inflammatory responses [21,22]. Figure 2d and Supplementary Figure S1b shows that the phosphorylation of STAT3, STAT6, and AKT was elevated in M2-polarized macrophages and was further enhanced by co-treatment with HK-HY449 and HK-HY9012.

### 3.3. HK-HY449 and HK-HY9012 Suppress Osteoclast Differentiation by Modulating Osteoclastogenic Gene Expression and MAPK Signaling Pathways

**Figure 3:**
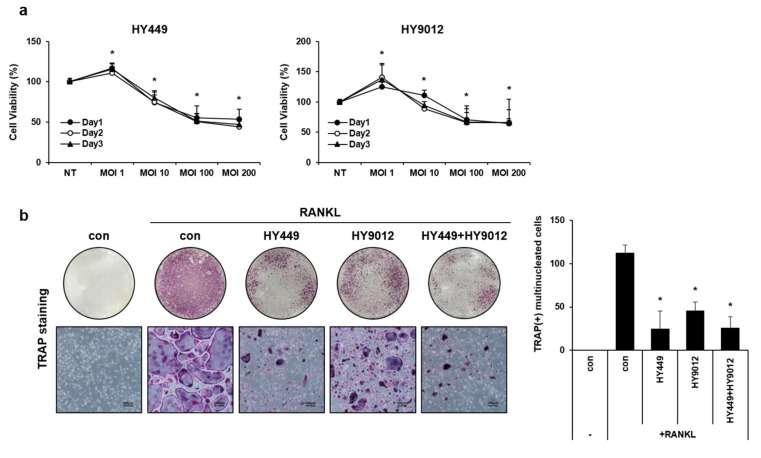
Effect of HK-HY449 and HK-HY9012 on cell viability and osteoclast differentiation. (**a**) BMMs were incubated with various MOIs of HK-HY449 and HK-HY9012. Cell viability was determined by the MTT assay daily over a period of 3 days. (**b**) During osteoclast differentiation, HK-HY449 and HK-HY9012 were added each time RANKL was replenished every 2–3 days. After 5 days, TRAP staining was performed. The number of TRAP-positive multinucleated cells was quantified. All quantitative data are presented as mean ± standard deviation; * *p* < 0.05 vs. control.

**Figure 4:**
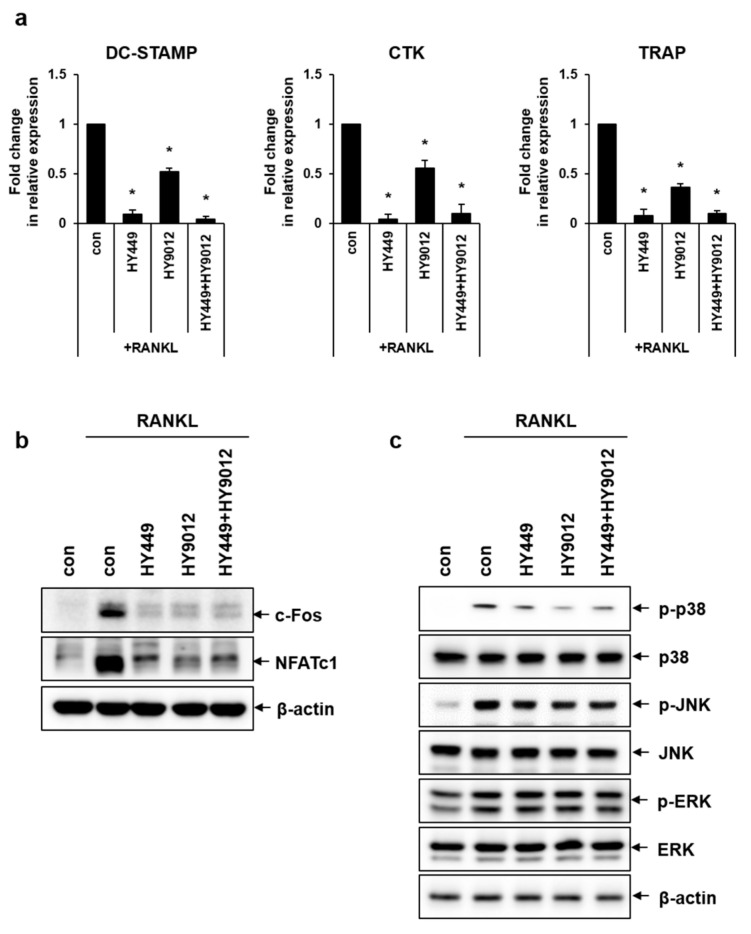
Effect of HK-HY449 and HK-HY9012 on expression of osteoclast-related genes and signaling pathways. BMMs were incubated in osteoclastogenic medium with HK-HY449 and HK-HY9012 for 5 days. (**a**) The mRNA levels of DC-STAMP, CTK, and TRAP were evaluated by real-time PCR. Expression of the control was set to 1, and values were normalized to β-actin mRNA. (**b**) Protein levels of c-Fos and NFATc1 were evaluated by Western blotting. (**c**) BMMs were treated with HK-HY449 and HK-HY9012 in osteoclastogenic medium for 10 min. The cell lysate was analyzed by Western blotting to detect phosphorylated p38, ERK, and JNK. Anti-β-actin was used as loading control. All quantitative data are presented as mean ± standard deviation; * *p* < 0.05 vs. control groups.

To investigate the effect of HK-HY449 and HK-HY9012 on osteoclast differentiation, BMMs were cultured in the presence of HK-HY449 or HK-HY9012 during the differentiation process. Prior to that, the viabilities of HK-HY449 and HK-HY9012 were assessed. BMMs were exposed to varying MOIs and cell viability was evaluated using the MTT assay over a period of 3 days (Figure 3a). Neither HK-HY449 nor HK-HY9012 showed notable cytotoxicity at MOI = 1, whereas both exhibited cytotoxic effects at higher MOI. Subsequent experiments were conducted at MOI = 1 to minimize potential cytotoxic effects. For osteoclast differentiation, BMMs were cultured for 5 days in the presence of HK-HY449, HK-HY9012, or both. The extent of osteoclast differentiation was assessed by TRAP staining, and the number of multinucleated TRAP-positive cells was quantified (Figure 3b). HK-HY449 significantly inhibited RANKL-induced osteoclast differentiation, whereas HK-HY9012 exhibited a relatively modest inhibitory effect. Notably, co-treatment with HK-HY449 and HK-HY9012 suppressed osteoclast differentiation to a level similar to that observed with HK-HY449 alone. To identify the molecular mechanisms underlying osteoclast differentiation and function, the effect of HK-HY449 and HK-HY9012 on the expression of osteoclast-specific TRAP, CTK, and DC-STAMP genes was examined. TRAP and CTK are well-established osteoclastogenic markers that contribute to the formation of mature osteoclasts [23], whereas DC-STAMP plays a pivotal role in osteoclast cell–cell fusion, a critical step in osteoclast maturation [24]. As expected, RANKL stimulation significantly upregulated the transcription of these genes. Instead, treatment with HK-HY449 or HK-HY9012 alone significantly suppressed it, and co-treatment maintained this suppressive effect at a similar level (Figure 4a). We next examined the protein expression of two master transcriptional regulators of osteoclast differentiation, c-Fos and NFATc1, which function downstream of RANKL and are essential for the transcriptional activation of osteoclast-specific genes [25,26]. Treatment with either HK-HY449 or HK-HY9012 alone markedly reduced RANKL-induced c-Fos and NFATc1 expression, and co-treatment resulted in a similar level of suppression (Figure 4b, Supplementary Figure S2a). To investigate upstream signaling events, we analyzed the phosphorylation status of MAPKs, including p38, JNK, and ERK, which mediate the RANKL-induced activation of c-Fos and NFATc1 [27,28,29]. RANKL stimulation led to strong phosphorylation of all three MAPKs. Treatment with HK-HY449 and HK-HY9012 reduced p38 MAPK and JNK phosphorylation, whereas ERK phosphorylation remained relatively unaffected (Figure 4c, Supplementary Figure S2b).

### 3.4. HK-HY449 and HK-HY9012 Attenuate Alveolar Bone Loss and Inflammation in a Ligature-Induced Periodontitis Model

**Figure 5:**
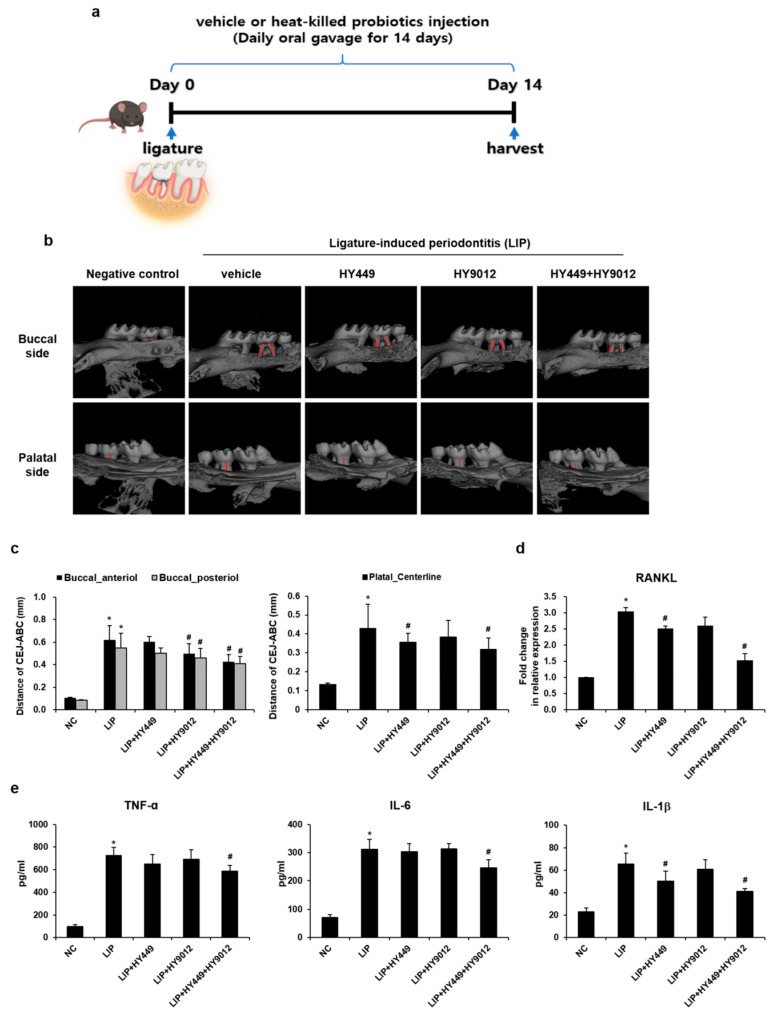
Effect of HK-HY449 and HK-HY9012 on ligature-induced periodontitis in an in vivo mouse model. Ligatures were placed around the left maxillary second molars, and mice received daily oral gavage of HK-HY449, HK-HY9012, or their combination for 14 days. Maxillae were collected on day 14. (**a**) Schematic timeline of ligature-induced periodontitis and probiotic administration. (**b**) Representative micro-computed tomography images showing vertical bone loss around the maxillary second molars after 14 days of ligation. (**c**) Alveolar bone loss was quantified by measuring the distance between the cementoenamel junction and the alveolar bone crest (CEJ–ABC) at the anterior and posterior buccal regions and the palatal centerline of the second molar using 3D reconstructions. (**d**) Relative mRNA expression levels of RANKL in gingival tissue, analyzed by RT-qPCR and normalized to β-actin. (**e**) Serum concentrations of TNF-α, IL-6, and IL-1β were measured by ELISA. All quantitative data are presented as mean ± standard deviation; * *p* < 0.05 vs. control, ^#^ *p* < 0.05 vs. ligature-induced periodontitis.

The ligature-induced periodontitis model consistently reproduced the key pathological features of human periodontal disease, including inflammation and alveolar bone loss [30]. The model was employed to evaluate the therapeutic potential of HK-HY449 and HK-HY9012. As shown in a schematic diagram (Figure 5a), periodontitis was induced by placing a ligature around the right maxillary second molar, followed by daily oral administration of HK-HY449, HK-HY9012, or their combination for 14 days. Micro-computed tomography confirmed that ligation led to a pronounced reduction in alveolar bone height around the maxillary second molar, with the most severe bone loss observed in the vehicle-treated group. While the individual administration of HK-HY449 or HK-HY9012 had minimal effects, their combined addition had a strong protective impact, significantly mitigating alveolar bone loss compared to either treatment alone (Figure 5b). Consistently, the CEJ–ABC distance, an established quantitative metric of periodontal destruction, was significantly increased in the vehicle group but was substantially reduced by HK-HY449 and HK-HY9012 co-treatment (Figure 5c). RT-qPCR analysis of gingival tissues revealed significant upregulation of RANKL following ligation-induced periodontitis, reflecting its role in bone resorption. This increase was markedly attenuated by co-treatment with HK-HY449 and HK-HY9012 (Figure 5d). Furthermore, to assess systemic inflammatory responses, serum levels of TNF-α, IL-6, and IL-1β were analyzed. These pro-inflammatory cytokines were elevated in the vehicle group but declined significantly upon co-treatment with HK-HY449 and HK-HY9012 (Figure 5e). Consistent with the ELISA results, RT-qPCR analysis of gingival tissues showed a similar reduction in TNF-α, IL-6, and IL-1β expression (Supplementary Figure S3).

## 4. Discussion

Recent advances in next-generation sequencing have uncovered an imbalance in the composition and function of host microbiota, a condition commonly known as dysbiosis [31]. Experimental and clinical studies have indicated that dysbiosis of the oral microbiota may play a crucial role in the development of periodontitis [32]. Periodontal disease-induced dysbiosis is not confined to the oral cavity, but can translocate to the gastrointestinal tract, disrupting the gut microbiome balance and contributing to various systemic diseases [33,34,35]. Importantly, the interconnection between oral and gut dysbiosis represents a bidirectional pathway, because gut microbiota may also contribute to the development of periodontitis [36]. Probiotics have garnered attention for their potential positive influence on oral and gut microbiota [37,38]. In particular, heat-killed probiotics offer immunomodulatory and barrier-protective effects comparable to those of live strains, without the safety concerns associated with bacterial translocation or antibiotic resistance gene transfer [39]. Recently, heat-killed *Lacticaseibacillus paracasei* has been found to exert a significant anti-osteoporotic effect by modulating the gut microbiota [40]. In addition, heat-killed probiotics, such as *Lactobacillus salivarius* subsp. *salicinius* and *Lactobacillus paracasei*, can enhance the oral microbiota by favoring beneficial populations and potentially inhibiting pathogenic bacteria associated with periodontitis [41]. Based on these observations, future studies should determine whether heat-inactivated *L. lactis* HY449 or *S. thermophilus* HY9012 modulate oral and gut dysbiosis in an in vivo periodontitis model.

Probiotics, bisphosphonates, nonsteroidal anti-inflammatory drugs, and statins are considered host modulatory agents for the treatment of periodontal diseases [42,43]. Host modulation therapy in the context of periodontitis is an advanced therapeutic approach that seeks to manage periodontal disease by altering the host immune and inflammatory responses rather than targeting solely bacterial infections [44,45]. Recent studies have suggested that probiotics, including their heat-killed forms, may serve as effective host-modulating agents for the management of periodontitis [46,47,48]. The potential mechanisms by which probiotics could alleviate the pathogenesis of periodontitis in vitro and in preclinical animal studies include modulation of the balance between pro-inflammatory and anti-inflammatory responses, regulation of T cell activity, enhancement of mucosal barrier function, and regulation of bone metabolism [3,49] Heat-killed *Lactobacillus plantarum* L-137 modulated the host immune response, enhancing Th1-related immune functions, and potentially reduced probing depth in periodontitis patients [50]. In this study, we demonstrated that HK-HY449 and HK-HY9012 regulated the polarization of macrophages, reduced osteoclastogenesis in vitro, and improved periodontal inflammation in an experimental periodontitis animal model. In addition, we observed that HK-HY449 and HK-HY9012, especially in combination, further reduced the expression of MMP-3, MMP-13, hs-CRP, and RANKL in human periodontal ligament fibroblasts under inflammatory conditions, suggesting a synergistic anti-inflammatory effect (Supplementary Figure S4). These findings are consistent with the results observed in M1/M2 macrophages and the in vivo periodontitis model, further strengthening the mechanistic relevance and therapeutic potential of the combined formulation. Future studies are warranted to evaluate whether HK-HY449, HK-HY9012, or their combination could serve as an adjunctive treatment for patients with periodontitis and to clarify the underlying mechanisms of action.

Recent investigations have revealed the significant influence of M1 and M2 macrophage phenotypes on the regulation of osteoclastogenesis and subsequent alveolar bone resorption [8]. The ratio of M1 to M2 macrophages correlates positively with the severity of periodontal diseases, which is associated with the expression of pro-inflammatory cytokines IL-1β and MMP-9 in gingival tissues [5,6]. Elevated levels of M1 macrophages within periodontal tissues correlated positively with enhanced alveolar bone resorption in an animal model of periodontitis [5]. Macrophage polarization toward the M2 phenotype prevents alveolar bone loss in mouse periodontitis [51]. Here, the combination of *L. lactis* and *S. thermophilus* reduced *P. gingivalis* LPS-induced polarization of M1 macrophages. These findings further indicate that the administration of postbiotic *L. lactis* or *S. thermophilus* has the potential to modulate the M1/M2 macrophage polarization ratio within periodontal tissues of an in vivo ligature-induced periodontitis model.

## 5. Conclusions

In conclusion, our study demonstrates that HK-HY449 and HK-HY9012 modulate inflammatory responses and bone resorption in periodontitis. By demonstrating their capacity to modulate osteoclast differentiation and macrophage polarization, we identified potential therapeutic mechanisms that may complement conventional treatment strategies for periodontitis. However, further studies are needed to validate the efficacy and safety of HK-HY449 and HK-HY9012 in humans through well-controlled clinical trials. Additionally, the effect of postbiotic treatment on the composition of the oral microbiota was not assessed in this study, which may be important for understanding its long-term clinical outcomes.

## Data Availability

The data presented in this study are available upon request from the corresponding author.

## Supplementary Materials

The following supporting information can be downloaded at: https://www.mdpi.com/article/10.3390/nu17162638/s1, Figure S1: Densitometric analysis of polarization-associated signaling proteins in M1 and M2 macrophages.; Figure S2: Densitometric analysis of osteoclast-related proteins and signaling molecules.; Figure S3: Effect of HK-HY449 and HK-HY9012 on gingival tissues in a mouse model of ligature-induced periodontitis.; Figure S4: Effects of HK-HY449 and HK-HY9012 on inflammatory gene expression in periodontal ligament fibroblasts stimulated with *P. gingivalis* LPS.; Table S1: Primer sequences for real-time PCR.

## Abbreviations

The following abbreviations are used in this manuscript:

BMM	Bone marrow-derived macrophages
CTK	Cathepsin K
DC-STAMP	Dendritic cell-specific transmembrane protein
ELISA	Enzyme-linked immunosorbent assay
ERK	Extracellular signal-regulated kinase
IFN	Interferon
IL	Interleukin
JNK	c-Jun N-terminal kinase
LPS	Lipopolysaccharide
M-CSF	Macrophage colony-stimulating factor
MAPK	Mitogen-activated protein kinase
MOI	Multiplicity of infection
NFATc1	Nuclear factor of activated T-cells cytoplasmic 1
PBS	Phosphate-buffered saline
PMA	Phorbol 12-myristate 13-acetate
RANKL	Receptor activator of nuclear factor κB ligand
RT-qPCR	Reverse transcription-quantitative PCR
TNF-α	Tumor necrosis factor-α
TRAP	Tartrate-resistant acid phosphatase
