# Supplementary material for: Postbiotics Derived from Lactococcus lactis and Streptococcus thermophilus Attenuate Experimental Periodontitis by Modulating Macrophage Polarization and Osteoclastogenesis
Source: Nutrients. 2025 Aug 14;17(16):2638. doi: 10.3390/nu17162638 (PMC12388978; doi:10.3390/nu17162638)
Supplement: Supplementary file 1 [file nutrients-17-02638-s001.zip › nutrients-3789022-supplementary.pdf]

Postbiotics Derived from *Lactococcus lactis* and *Streptococcus thermophilus* Attenuate Experimental Periodontitis by Modulating Macrophage Polarization and Osteoclastogenesis

Hyun-Joo Park [1,2,†], Mi-Kyoung Kim [1,†], Soon Chul Heo [1], Dong Ki Hong [3], Soo-Dong Park [3], Yeon Kim [1,2], Soo-Kyung Bae [2,4], Hyung Joon Kim [1,2] and Moon-Kyoung Bae [1,2,*]

1 Department of Oral Physiology, School of Dentistry, Pusan National University, Yangsan 50612, Republic of Korea
2 Dental and Life Science Institute, School of Dentistry, Pusan National University, Yangsan 50612, Republic of Korea
3 R&BD Center, hy Co., Ltd., Yongin-si 17086, Republic of Korea
4 Department of Dental Pharmacology, School of Dentistry, Pusan National University, Yangsan 50612, Republic of Korea
* Correspondence: mkbae@pusan.ac.kr
† These authors contributed equally to this work.

Supplementary Information

The following file provides supplementary data for the manuscript titled "Postbiotics derived from *Lactococcus lactis* and *Streptococcus thermophilus* Attenuate Experimental Periodontitis by Modulating Macrophage Polarization and Osteoclastogenesis".

The following supplementary materials are provided in this file:

• Supplementary Table S1

• Supplementary Figures S1−S4 and their corresponding legends

Supplementary Table

Table S1. Primer sequences for real-time PCR

	Genes	Sequences (5´→ 3´)
Mouse	β-actin	Forward: TGTTACCAACTGGGACGACA<br>Reverse: GGGGTGTTGAAGGTCTCAAA
	TNF-α	Forward: GGTGCCTATGTCTCAGCCTCTT<br>Reverse: GCCATAGAACTGATGAGAGGGAG
	IL-6	Forward: TGGTGACAACCACGGCCTTCR<br>Reverse: GCCTCCGACTTGTGAAGTGGT
	IL-1β	Forward: TGCTGGTGTGTGACGTTCCC<br>Reverse: GTCCGACAGCACGAGGCTTT
Human	β-actin	Forward: ACTCTTCCAGCCTTCCTTCC<br>Reverse: TGTTGGCGTACAGGTCTTTG
	MMP3	Forward: CACTCACAGACCTGACTCGGTT<br>Reverse: AAGCAGGATCACAGTTGGCTGG
	MMP13	Forward: CCTTGATGCCATTACCAGTCTCC<br>Reverse: AAACAGCTCCGCATCAACCTGC
	hs-CRP	Forward: GTCTTGACCAGCCTCTCTCA<br>Reverse: GTCGAGGACAGTTCCGTGTA
	RANKL	Forward: ATGCGGTTTGCAGTTCTT<br>Reverse: CTCACTCCTTATCTCCACTTAGG

Supplementary Figures

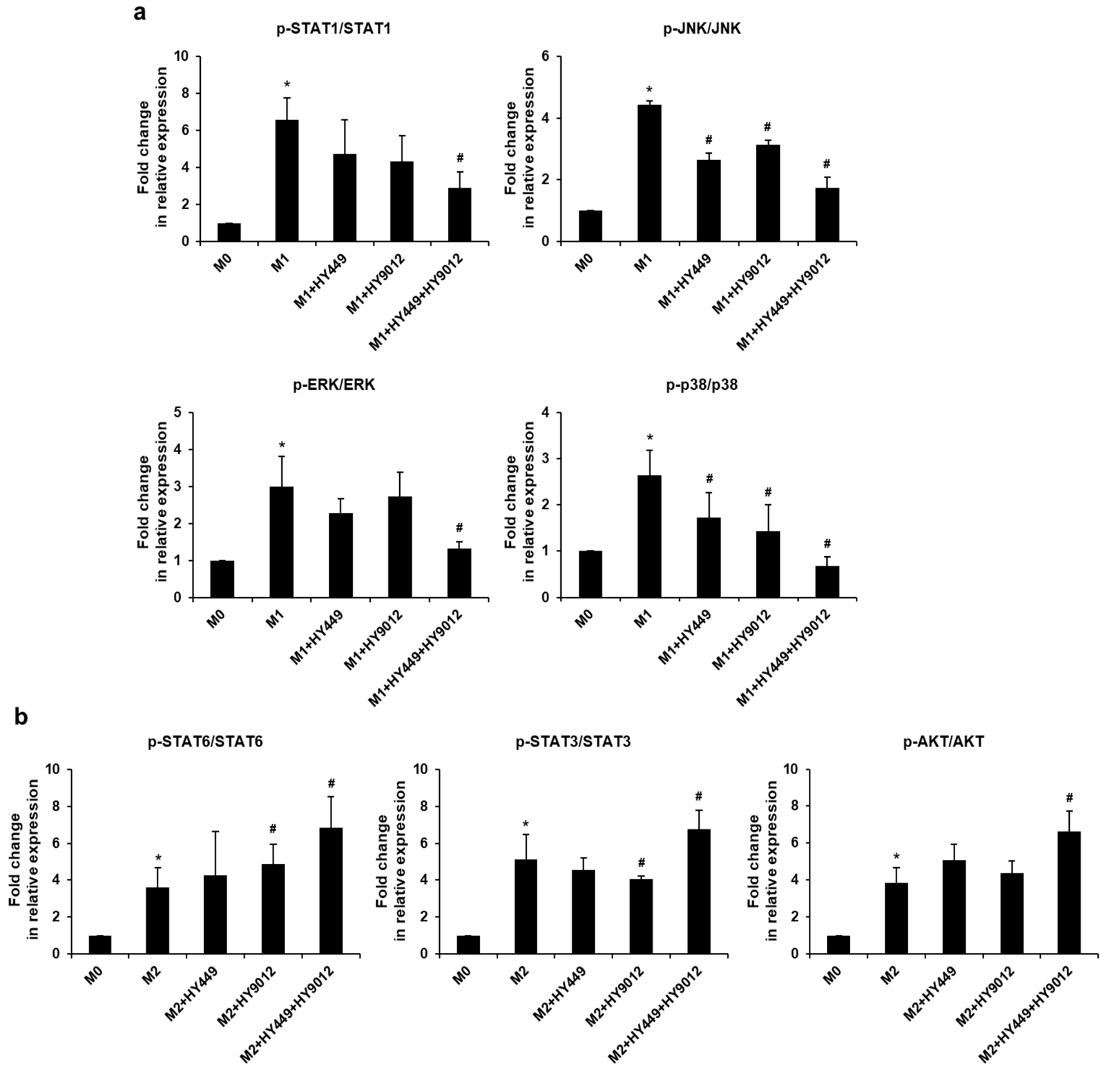

Figure S1. Densitometric analysis of polarization-associated signaling proteins in M1 and M2 macrophages. Western blot analysis was performed to evaluate the expression levels of phosphorylated STAT1, JNK, ERK, and p38 in M1 macrophages **(a)**, and phosphorylated STAT6, STAT3, and AKT in M2 macrophages **(b)**. Phosphorylated proteins were normalized to their respective total protein levels (STAT1, JNK, ERK, p38, STAT6, STAT3, and AKT). Data are presented as mean ± SEM from three independent experiments. $^{*}P < 0.05$ vs. M0, $^{\#}P < 0.05$ vs. M1.

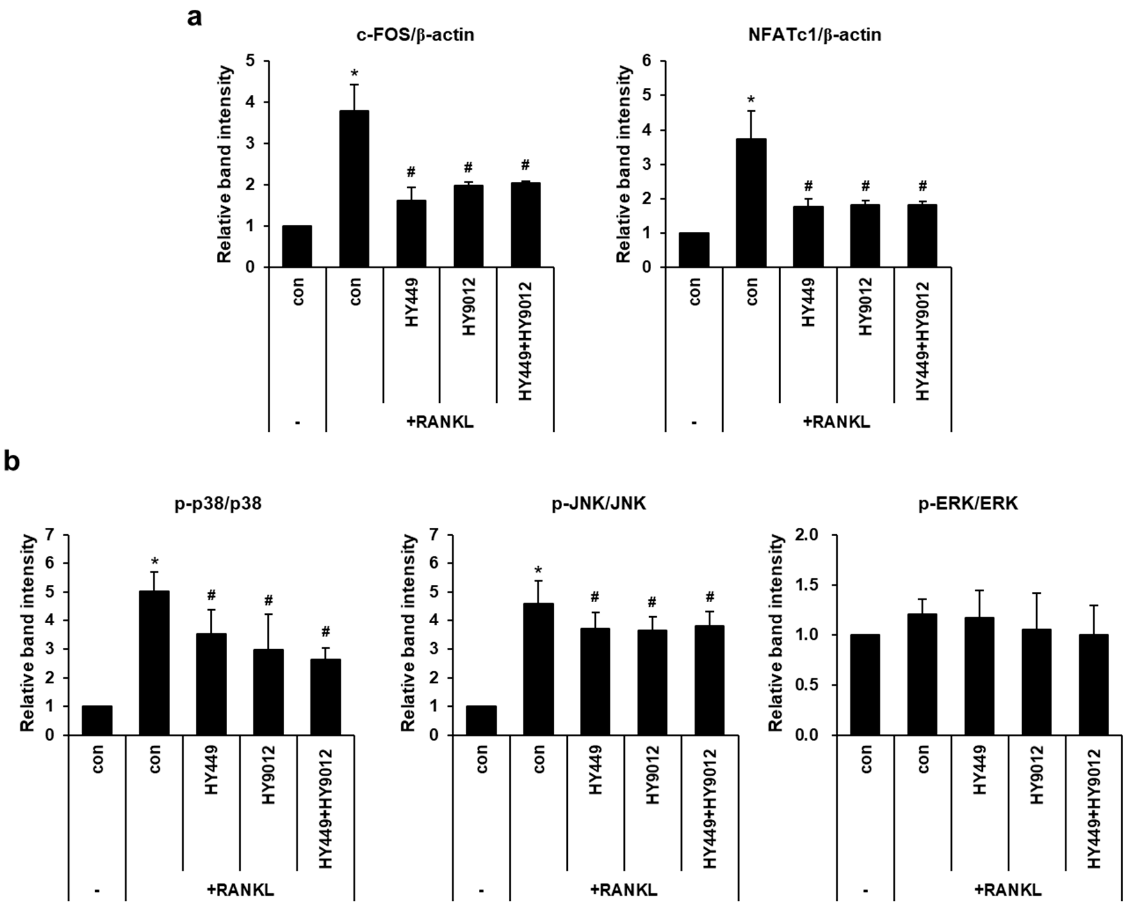

Figure S2. Densitometric analysis of osteoclast-related proteins and signaling molecules. Western blot analysis was performed to evaluate the expression levels of c-Fos, NFATc1, and phosphorylated forms of p38, ERK, and JNK. **(a)** Protein levels of c-Fos and NFATc1 were normalized to β-actin, which served as a loading control. **(b)** Phosphorylated MAPKs were normalized to their corresponding total protein levels. Data are presented as mean ± standard deviation from three independent experiments. $^{*}P < 0.05$ vs. control groups.

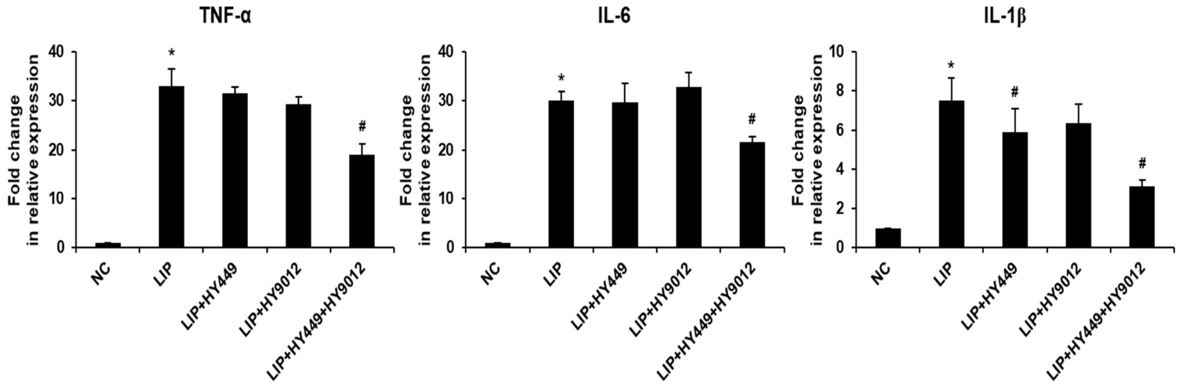

Figure S3. Effect of HK-HY449 and HK-HY9012 on gingival tissues in a mouse model of ligature-induced periodontitis. Ligatures were placed around the left maxillary second molars, and mice received daily oral gavage of HK-HY449, HK-HY9012 or their combination for 14 days. Maxillae were collected on day 14. Total RNA was extracted from gingival tissues, and relative mRNA expression levels of TNF-α, IL-6, and IL-1β were analyzed by RT-qPCR and normalized to β-actin. All quantitative data are presented as mean ± standard deviation; $^{*}P < 0.05$ *vs*. control, $^{\#}P < 0.05$ *vs*. ligature-induced periodontitis.

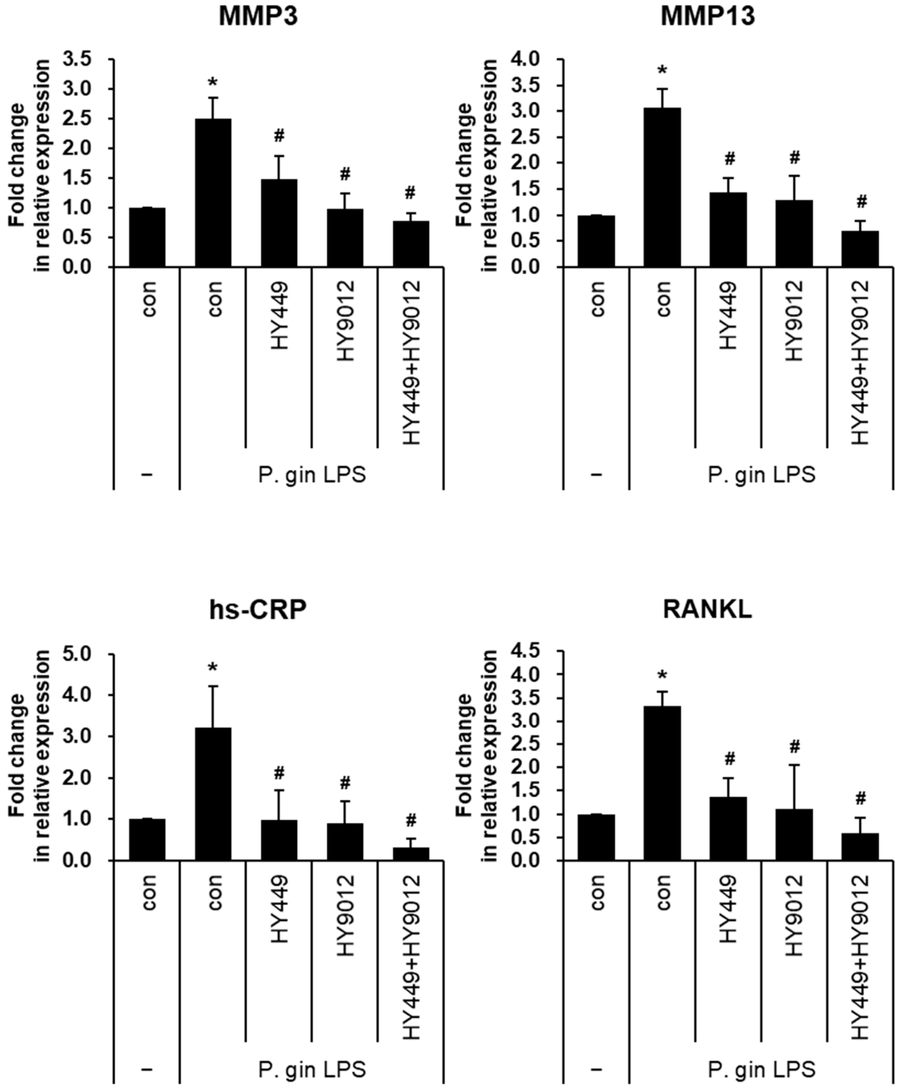

Figure S4. Effects of HK-HY449 and HK-HY9012 on inflammatory gene expression in periodontal ligament fibroblasts stimulated with *P. gingivalis* LPS. Periodontal ligament fibroblasts were treated with 1 μg/mL *P. gingivalis* LPS (*P. gin* LPS) and subsequently incubated with HK-HY449 and HK-HY9012, or a 1:1 mixture of both strains for 8 h. (a) The mRNA levels of MMP3, MMP13, hs-CRP, and RANKL were evaluated by real-time PCR. Expression of the control was set to 1, and values were normalized to β-actin mRNA. All quantitative data are presented as mean ± standard deviation; *P < 0.01 *vs*. control, #P < 0.01 *vs*. *P. gin* LPS.
